# Impact of ChAdOx1 or DNA Prime Vaccination on Magnitude, Breadth, and Focus of MVA-Boosted Immunogen-Specific T Cell Responses

**DOI:** 10.3390/vaccines12030279

**Published:** 2024-03-07

**Authors:** Alex Olvera, Luis Romero-Martin, Bruna Oriol-Tordera, Miriam Rosas-Umbert, Tuixent Escribà, Beatriz Mothe, Christian Brander

**Affiliations:** 1 Irsicaixa—AIDS Research Institute, 08916 Badalona, Barcelona, Spain; luis.romero-martin@pasteur.fr (L.R.-M.); tescriba@irsicaixa.es (T.E.); bmothe@irsicaixa.es (B.M.); cbrander@irsicaixa.es (C.B.); 2 Faculty of Sciences and Technology, University of Vic-Central University of Catalonia (UVic-UCC), 08500 Vic, Barcelona, Spain; 3 CIBER de Enfermedades Infecciosas (CIBERINFEC), Instituto de Salud Carlos III, 28029 Madrid, Spain; 4 Department of Infectious Diseases, Hospital Germans Trias I Pujol (HUGTIP), 08916 Badalona, Barcelona, Spain; 5 Fundació Lluita contra les Infeccions, Hospital Germans Trias I Pujol (HUGTIP), 08916 Badalona, Barcelona, Spain; 6 Department of Infectious Diseases and Immunity, University of Vic-Central University of Catalonia (UVic-UCC), 08500 Vic, Barcelona, Spain; 7 Institució Catalana de Recerca i Estudis Avançats (ICREA), 08010 Barcelona, Spain

**Keywords:** T-cell vaccine, HIV, immunodominance, immune memory

## Abstract

The efficacy of anti-viral T-cell vaccines may greatly depend on their ability to generate high-magnitude responses targeting a broad range of different epitopes. Recently, we created the HIV T-cell immunogen HTI, designed to generate T-cell responses to protein fragments more frequently targeted by HIV controllers. In the present study, we aim to maximize the breadth and magnitude of the T-cell responses generated by HTI by combining different vaccine vectors expressing HTI. We evaluated the ability to induce strong and broad T-cell responses to the HTI immunogen through prime vaccination with DNA plasmid (D) or Chimpanzee Adenovirus Ox1 (ChAdOx1; C) vectors, followed by a Modified Virus Ankara (MVA; M) vaccine boost (DDD, DDDM, C, and CM). HTI-specific T-cell responses after vaccination were measured by IFN-γ-ELISpot assays in two inbred mice strains (C57BL/6 and BALB/c). CM was the schedule triggering the highest magnitude of the response in both mice strains. However, this effect was not reflected in an increase in the breadth of the response but rather in an increase in the magnitude of the response to specific immunodominant epitopes. Immunodominance profiles in the two mouse strains were different, with a clear dominance of T-cell responses to a Pol-derived peptide pool after CM vaccination in C57BL/6. Responses to CM vaccination were also maintained at higher magnitudes over time (13 weeks) compared to other vaccination regimens. Thus, while a ChAdOx1 prime combined with MVA booster vaccination generated stronger and more sustained T-cell responses compared to three DNA vaccinations, the ChAdOx1 primed responses were more narrowly targeted. In conclusion, our findings suggest that the choice of vaccine vectors and prime-boost regimens plays a crucial role in determining the strength, duration, breadth, and focus of T-cell responses, providing further guidance for selecting vaccination strategies.

## 1. Introduction

Vaccines have helped to greatly reduce the burden caused by infectious diseases on human health in the past and even to eradicate one human viral infection, smallpox [1,2]. Unfortunately, the development of a preventive vaccine against the human immunodeficiency virus (HIV) has proven to be challenging and, 40 years after the identification of HIV, no prophylactic vaccine has become available. HIV’s high mutational rate, enabling rapid immune escape, its capacity to create a latent viral reservoir and to impair the immune system, together with the lack of well-defined functional immune correlates of protection from infection, have thwarted all attempts to develop an effective vaccine against HIV [2,3,4].

The identification of immune correlates has been difficult due to the few reports of a natural cure for HIV infection [5,6], none providing evidence of immune-mediated viral clearance. Still, a group of less than 1% of people with HIV (PWH), usually named HIV elite-controllers, are capable of suppressing viremia to undetectable levels, maintaining high CD4 counts, in the absence of antiretroviral therapy (ART) [7,8]. Another minority of PWH, referred to as HIV viral controllers, can maintain low viral loads (generally below 2000 copies/mL) and stable CD4 counts in the absence of ART for years [7,9,10]. Many research efforts have been devoted to the study of the immune response of elite and viral controllers, to develop a vaccine that could protect from infection or mediate low viral loads in breakthrough infections. In the therapeutic setting, these vaccines could provide support for cure strategies aimed at controlling or eliminating HIV from infected individuals [11].

There are several studies supporting the important role of T-cells in HIV infection control: (i) associations between specific human leukocyte antigen class I (HLA-I) alleles and relative control of infection [12,13,14], (ii) the presence of HIV mutations associated with certain HLA class I alleles referred to as HLA footprints [15,16,17,18], (iii) the emergence of HIV-specific CD8+ T-cells coinciding with viral set-point onset [19], and (iv) CD8+ T-cell depletion in animal studies that lead to the loss of viral control [4,20]. Additionally, T-cell responses would have to focus on especially vulnerable regions of the HIV proteome, where mutation occurs at high viral replication capacity cost, to avoid HIV immune escape [21,22,23]. More recently, virus control in the SIV infection model was observed after passively administrated neutralizing antibodies that boosted effective CD8+ T-cell immunity able to mediate long-term control over virus replication [24].

This concept has been reflected in the development of several HIV immunogens, usually targeting highly conserved regions of the HIV genes, to avoid viral escape [25,26,27,28,29,30,31,32,33,34,35,36,37,38,39,40,41,42,43,44,45,46,47,48,49,50,51,52,53,54]. However, our group has designed a T-cell immunogen that is informed by a detailed analysis of the cellular immune responses to HIV of more than 1000 PWH [55,56]. Using these data, we have identified the targets of the HIV-specific T-cell response in elite and viral controllers and defined regions preferentially targeted in these populations [57]. Based on these regions, we designed the HIV T-cell Immunogen (HTI) to target T-cell responses to HIV proteome fragments vulnerable to T-cell immunity, avoiding responses to decoy epitopes [56]. HTI has been shown to be immunogenic in mice and non-human primates [56,58,59] when using different vaccine vectors such as DNA plasmids, Chimpanzee Adenovirus (ChAdOx1), Modified Vaccinia virus Ankara (MVA), or Bacillus Calmette–Guérin (BCG), and has been successfully tested in human clinical trials of therapeutic HIV vaccination [60].

The objective of the experiments reported herein was to explore the capacity of HTI to generate strong, long, and broadly distributed T-cell responses using prime-boost strategies which have shown promise in other vaccine settings [61,62]. In previous studies, we have found that prime vaccination of three immunizations with DNA plasmids expressing HTI (DNA.HTI or D), followed by a boost with an MVA vector also expressing HTI (MVA.HTI or M), induce the highest magnitude of response in murine spleen cells [56]. This regimen was also adopted in the first phase of the AELIX-002 clinical trial, where it again showed strong immunogenicity in PWH [60]. Here, we aimed to further increase the magnitude of vaccine-induced T-cell responses and to simplify the vaccination regimen by using a ChAdOx1 vector expressing HTI (ChAdOx1 or C) as the prime vaccination for an MVA.HTI boost. We also wanted to probe whether the same vaccine regimen in two mouse strains (C57BL/6 and BALB/c) with different major histocompatibility complex alleles would induce responses of comparable magnitude and immunodominance patterns.

## 2. Materials and Methods

### 2.1. Vaccines

The HIVACAT T-cell immunogen (HTI) was used in all vaccinations. Briefly, this 529-amino-acid immunogen was designed as the concatenation of 16 HIV protein fragments that are preferentially targeted by T-cells from HIV-infected individuals showing natural control of HIV infection [56]. The resulting amino acid sequence was retrotranscribed into nucleotides and the resulting open reading frame (ORF) was inserted into different vectors [56]: a DNA plasmid under the control of a CMV promoter (DNA.HTI, D) and two viral vectors: Chimpanzee Adenovirus Ox1 (ChAdOx1.HTI, C) and Modified Vaccinia Ankara (MVA.HTI, M). In this study, we used endotoxin-free DNA.HTI vaccine stocks (Plasmid factory, Bielefeld, Germany) and ChAdOx1.HTI produced in HEX293A T-REx^®^ cells (ThermoFisher Scientific, Inc., Waltham, MA, USA), as well as MVA.HTI produced in chicken embryo fibroblasts kindly provided by Dr T. Hanke’s laboratory at Oxford University.

### 2.2. Mice and Immunization Regimens

**Figure 1:**
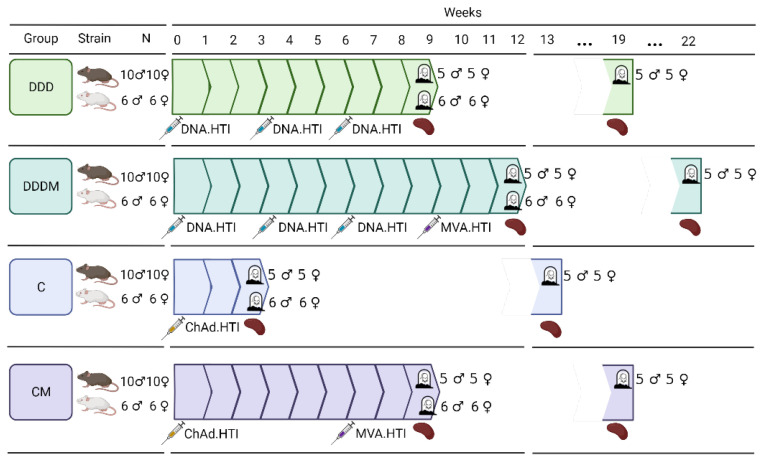
Vaccination schedule. The number of animals used, the week of each immunization, and the week of post-immunization (wpi) sampling are indicated for each vaccination regimen. C57BL/6 mice are represented by black and BALB/c mice by white. Created using Biorender.com.

Six-week-old mice of two different strains, C57BL/6JOlaHsd (N = 80 females and 80 males) and BALB/cOlaHsd (N = 48 females and 48 males), were used in these experiments. Animals were purchased from ENVIGO (Kreuzelweg, The Netherlands) and housed in the level 3 biological containment unit at the Catalan Comparative Medicine and Bioimage Center (CMCiB) animal facility (Badalona, Spain), under controlled conditions (temperature 22 ± 2 °C, pressure −20 pascals, 12 h light/dark cycles). Mice were non-randomly divided into four experimental groups (10 males and 10 females per C57BL/6 group, 6 males and 6 females per BALB/c group), with a maximum of 5 animals per cage, and allowed to acclimate for one week before the initial treatment. Animals were open-label, intramuscularly immunized, with the dose split equally in both quadriceps (caudal thigh muscles). Each mouse strain was vaccinated with four different vaccination regimens, as shown in Figure 1.

Briefly, one group was primed with three DNA.HTI doses (100 µg/animal) spaced by three weeks (DDD), a second group received the three DNA.HTI prime followed by an MVA.HTI boost (10^6^ plaque-forming units/animal) after three weeks (DDDM), a third group received a prime with a single dose (10^9^ viral particles/animal) of ChAdOx1.HTI (C), and a fourth group received a ChAdOx1.HTI prime followed by an MVA.HTI boost six weeks apart (CM). Five female and male C57BL/6 mice and six female and male BALB/c mice per experimental group were euthanized three weeks post-last immunization (wpi). An additional five female and male C57BL/6 animals per experimental group were euthanized at 13 wpi to determine the maintenance of the vaccine-induced immune response.

### 2.3. Sample Processing

At the study’s final time point, the animals were euthanized and their spleens were recovered aseptically. Spleen cells were isolated after mechanical tissue disaggregation and passaged through a 40 μm cell strainer (Falcon—FisherScientific, Madrid, Spain) using a 5 mL rubber syringe plunger. Following red blood cell lysis with RBC lysis buffer (17 mM Tris and 0.14 M NH_4_Cl; Sigma-Aldrich Merck, Darmstadt, Germany), spleen cells were washed twice with R10 (RPMI 1640, supplemented with 10% decomplemented FBS, penicillin (100 U/mL), and streptomycin (100 µg/mL), Gibco ThermoFisher Scientific, Waltham, MA USA), and used to measure the T-cell immune response in an IFN-γ ELISPOT assay. Leftover cells were cryopreserved in FBS with 10% DMSO (Sigma-Aldrich Merck, Darmstadt, Germany) and stored in liquid N_2_ until use.

### 2.4. Overlapping Peptides Covering the Whole HTI Sequence

As T-cell recall antigen for in vitro stimulations, a set of 147 15-amino-acid overlapping peptides (OLP), spanning the entire HTI sequence with an 11-amino-acid (aa) overlap, was designed using the PeptGen algorithm in the Los Alamos HIV database [63]. To assess responses to HTI, OLPs were divided into 17 peptide pools according to the HIV protein subunit fragments they were derived from (Table S1): 7 for Gag (8–11 peptides/pool), 7 for Pol (11 or 5 peptides/pool), 2 for Vif (8 and 6 peptides/pool), and 1 for Nef (2 peptides/pool).

### 2.5. IFN-γ ELISPOT Assay

IFN-γ ELISPOT assays were performed using the mouse IFN-γ ELISPOT ALP kit (Mabtech, Nacka Strand, Sweden), following the manufacturer’s instructions with minor modifications. Live spleen cells were counted (NucleoCounter^®^ NC-3000, Chemometec, Allerod, Denmark) and cell density was adjusted with R10 to plate 4 × 10^5^ cells/well in 96-well polyvinylidene plates (Millipore—Merck, Darmstadt, Germany), previously coated with an IFN-γ capture antibody (clone AN-18). Cells were stimulated with the above-mentioned HTI-specific OLP pools (14 μg/mL final concentration for each peptide) for 16 h at 37 °C in 5% CO_2_. Concanavalin A (Sigma-Aldrich Merck, Darmstadt, Germany), at 5 µg/mL, was used as a positive control, and R10 alone as a negative control.

After overnight stimulation, plates were developed by adding a biotinylated IFN-γ detection antibody (clone R4-6A2), streptavidin conjugated with alkaline phosphatase (AP), and the AP conjugate substrate kit (Bio-Rad Laboratories, Madrid, Spain). Spot-forming cells (SFC) per well were counted using an automated ELISPOT reader system (Cellular Technology Limited Analyzers LLC, Cleveland, OH, USA) using the ImmunoSpot software v2.7.4 and adjusted for 10^6^ spleen cells. The threshold for positive responses for each animal was defined as the highest of the following three criteria: (i) a minimum of 50 SFC/10^6^ spleen cells, (ii) responses exceeding the mean SFC/10^6^ spleen cells in the negative control wells plus 3 standard deviations of the negative control wells, and (iii) three times the mean SFC/10^6^ spleen cells of the negative control wells. Cells from animals with backgrounds higher than 400 SFC/10^6^ spleen cells were considered overactivated and corresponding data were excluded. The magnitude of the response was expressed as SFC per million splenocytes and the mean of the negative wells for each animal was background-subtracted from each OLP pool-specific response. Pools showing responses below the threshold were considered non-responding pools and not summed in the total magnitude.

### 2.6. IFN-γ Response Mapping to Individual Peptides

Additional experiments were performed to map pool-specific responses to the specific OLP they contained, using cryopreserved samples of C57BL/6 and BALB/c mice immunized with DDDM. Initially, positive pools were deconvoluted to identify OLP-triggering positive responses by testing every peptide in a positive pool individually. Due to the need for a large number of cells, the spleen cells from animals in the same group were pooled for these experiments. These pooled spleen cells were stimulated overnight at 37 °C 5% CO_2,_ and IFN-γ-producing cells were identified using an ELISPOT assay (Mabtech), as previously described.

We also titrated the responses to the peptides triggering IFN-γ responses independently. To optimize the use of cells, if two overlapping OLPs triggered responses, we assumed that the shared responses targeted the common 11 amino acids and only titrated the OLP triggering the stronger response. Titration was performed by testing, in an IFN-γ ELISPOT assay, each reactive OLP at 20, 5, 1.25, 0.3, 0.08, and 0.02 µg/mL, using C57BL/6 spleen cells vaccinated with DDDM or CM. To calculate the IC50, titration results were adjusted to a non-linear regression using least squares fit. Only regressions with a goodness of fit R > 0.95 were accepted and reported.

### 2.7. Statistical Analysis

The results were analyzed using GraphPad software v10. Comparisons between two groups were performed with the non-parametric Mann–Whitney test. Comparisons among more than two groups were evaluated using the nonparametric Kruskal–Wallis test, correcting for multiple comparisons with the Dunn test. Comparisons between groups over time were performed using a two-way ANOVA, and multiple comparisons were tested with the Tukey test. Statistical significance was set at *p* < 0.05 and q < 0.01.

## 3. Results

### 3.1. ChAdOx1.HTI Is a Stronger T-Cell Prime Than Three DNA.HTI for an MVA.HTI Boost

Four different vaccination regimens (DDD, DDDM, C, and CM) were tested in two different mouse strains (C57BL/6 and BALB/c). Three (DDD, DDDM, C) of the four different vaccination regimens tested showed a similar background range in the negative control wells of the ELISpot assay. The exception was the CM regimen which caused an elevated unspecific T-cell activation in both strains (Figure S1). This initial observation is consistent with the prime-boost regimen using, in both cases, viral vaccine vectors, which may cause broader unspecific T-cell activation compared to prime vaccinations with DNA plasmids or a single viral vector immunization. To account for these differences, we performed background subtraction of the IFN-γ SFC data across all subsequent experiments.

**Figure 2:**
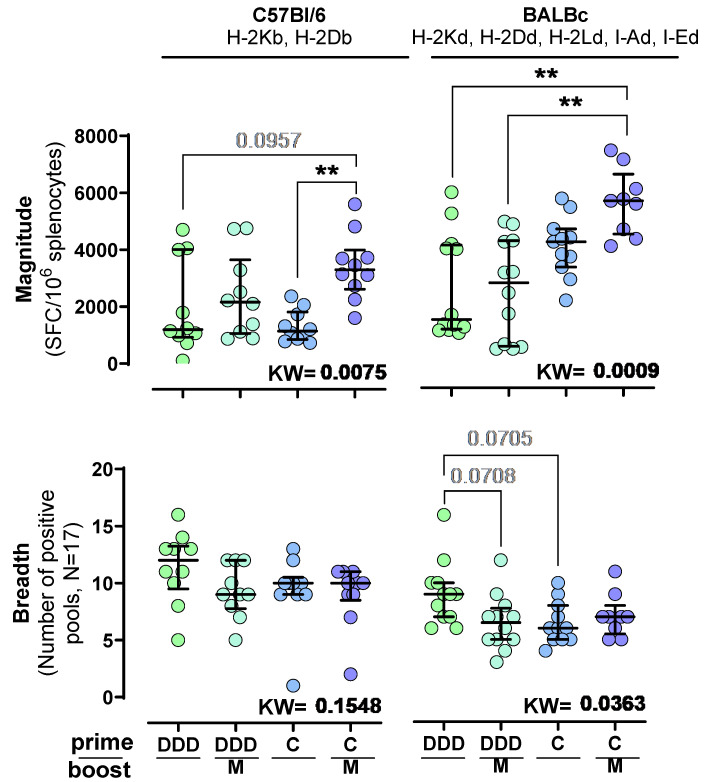
Magnitude and breadth of the T-cell response to HTI. INFγ SFC/10^6^ spleen cells and the number of reactive peptide pools, after stimulation with 17 pools containing OLPs covering the HTI sequence, in C57BL/6 and BALB/c mice vaccinated with four different prime-boost regimens (DDD, DDDM, C, and CM). Statistically significant differences (*p* < 0.05) between treatment regimens were tested with the Kruskal–Wallis test with Dunn’s correction for multiple comparisons. Significant adjusted Dunn’s *p*-values are shown as ** < 0.01. Trends (*p* < 0.1) are indicated by grey numbers.

Independently of the prime regimen (DDD or C), the MVA boost increased the median magnitude of IFN-γ HTI-specific responses and triggered the highest responses in both strains when using a C prime (CM median magnitude in C57BL/6 and BALB/c = 3298 and 5664 SFC/10^6^ spleen cells, respectively), although statistical significance was not achieved in either mouse strain. Briefly, the CM regimen response was higher compared to the DDD in BALB/c (Median magnitude = 1497 SFC/10^6^ spleen cells; adjusted Dunn *p* = 0.0034), DDDM in BALB/c (Median magnitude = 2789 SFC/10^6^ spleen cells; adjusted Dunn *p* = 0.0019), and C in C57BL/6 (Median magnitude = 1146 SFC/10^6^ spleen cells; adjusted Dunn *p* = 0.0052) regimens (Figure 2). Furthermore, responses were generally higher in BALB/c compared to C57BL/6, being statistically significant for the C and CM vaccination regimens (Mann–Whitney *p* < 0.0001 and 0.0010, respectively).

In both strains, the DDD prime promoted the broadest responses compared to the other immunization schedules (median breadth = 12 positive pools in C57BL/6 mice and nine in BALB/c mice). However, statistical significance was not reached.

### 3.2. ChAdOx1.HTI Prime Drives Different Immunodominance Compared to DNA.HTI Prime

**Figure 3:**
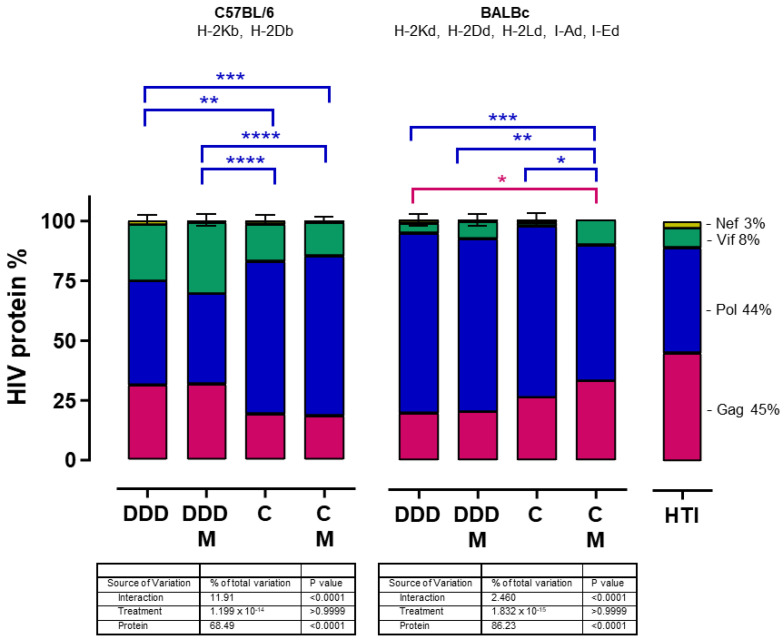
Distribution of the T-cell responses along the different HIV protein fragments that form HTI. The mean percentage of the total magnitude of the IFN-γ T-cell response to each of the four HIV proteins from which HTI fragments are derived is compared between C57BL/6 (**left**) and BALB/c (**middle**) mice and related to their proportions in the HTI sequence (**right**). Statistically significant differences were tested with a two-way ANOVA and multiple comparisons were performed with Tukey’s test. Sources of variation and the *p*-values of the different two-way ANOVA factors are indicated in the table below each graph. Statistically significant Tukey’s multiple comparisons between regimens for the different HTI protein fragments are indicated by colors (purple for Gag, Blue for Pol, Green for Vif, and yellow for Nef). Adjusted Tukey’s *p*-values are shown as * *p* < 0.05, by ** <0.01, by *** <0.001, and **** <0.0001.

We analyzed if the trend toward a lower breadth of responses in the CM immunization arm, despite reaching significantly higher magnitudes, was caused by the presence of immunodominant responses. To this end, we assessed whether the distribution of the magnitude of the T-cell response along the HTI sequence followed the percentual contribution of each HIV protein fragment (Gag 45%, Pol 44%, Vif 8%, and Nef 3%) to the HTI sequence and compared this distribution among different vaccination regimens. We found that the percentage of the total magnitude of Nef-, Vif-, Pol-, and Gag-specific responses after HTI vaccination was not a direct reflection of their percentage contribution to the immunogen composition, in both mouse strains and irrespective of the vaccination regimen. Furthermore, different vaccination regimens promoted different distributions of HTI-specific responses. In C57BL/6 mice, the DDD and DDDM regimens promoted more Gag and fewer Pol responses than the C and CM regimens. In BALBc mice, an opposite distribution was observed. The DDD regimen promoted less Gag responses than the CM regimen, while the CM regimen promoted less Pol responses than the DDD, DDDM, and C regimens (Figure 3).

**Figure 4:**
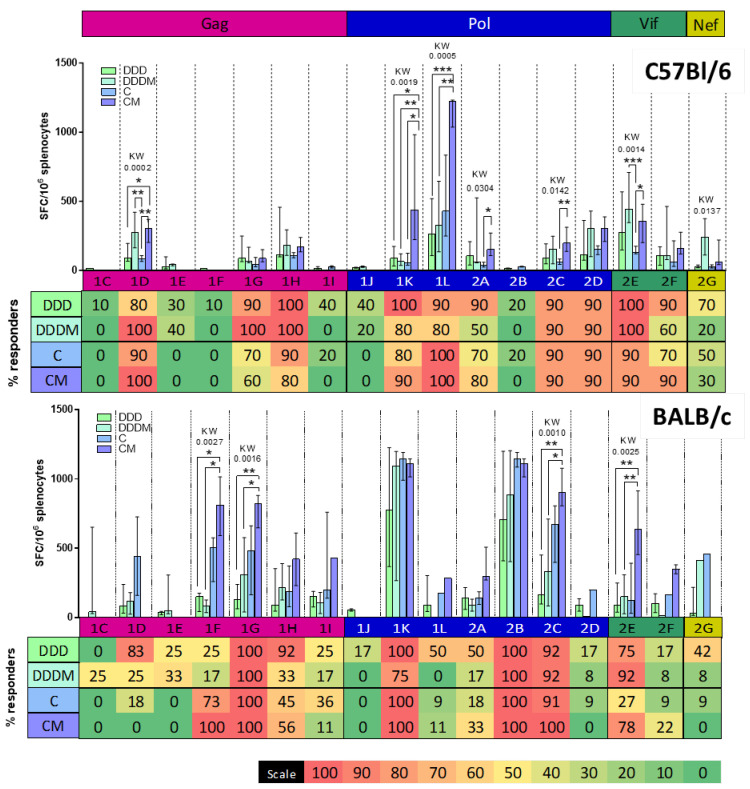
Focus of the response to HTI. The pool-by-pool magnitude of the response is shown for C57BL/6 mice (**up graph**) and BALB/c mice (**down graph**). The graph shows significant Kruskal–Wallis and adjusted Dunn’s *p*-values comparing the four vaccination regimens (DDD, DDDM, C, and CM) responses for each pool. Statistical significance for Dunn’s *p*-values is shown as * if *p* < 0.05 by, ** if <0.01, and by *** if <0.001. At the bottom of each graph, a heat map indicates the % of animals responding to each pool.

When comparing the magnitude of the response to the different pools (Figure 4), C57BL/6 showed dominant responses to two peptide pools in Pol, especially after CM vaccination 1K (median magnitude = 437 SFC/10^6^ spleen cells) and 1L (median magnitude = 1222 SFC/10^6^ spleen cells). Moreover, the pool 1D-specific (Gag) responses were boosted by an M vaccination after priming with DDD (median magnitude = 273 SFC/10^6^ spleen cells) or C (median magnitude = 302 SFC/10^6^ spleen cells), while a strong M booster effect after DDD was also seen towards pool 2E (Vif). Responses to these four pools (1K, 1L, 1D, and 2E) also generated responses in most of the animals (80–100% of the responders), making them consistent intra- and inter-individual immunodominant responses [64].

The analysis of pool-specific BALB/c responses showed a remarkably different profile: two pools in Pol triggered strong responses regardless of the regimen used, including pools 1K (median magnitude = 775, 1095, 1144, and 1112 SFC/10^6^ spleen cells for DDD, DDDM, C, and CM, respectively) and 2B (median magnitude = 707, 887, 1144, and 1112 SFC/10^6^ spleen cells for DDD, DDDM, C, and CM, respectively). Moreover, in BALB/c mice, the C prime triggered higher responses compared to DDD and DDDM in four additional pools, which were further increased by an M boost (Gag pools 1F and 1G, Pol pool 2C, and Vif pool 2E). Altogether, these pools were also the ones that triggered responses in almost all BALB/c mice, regardless of the regimen used.

**Figure 5:**
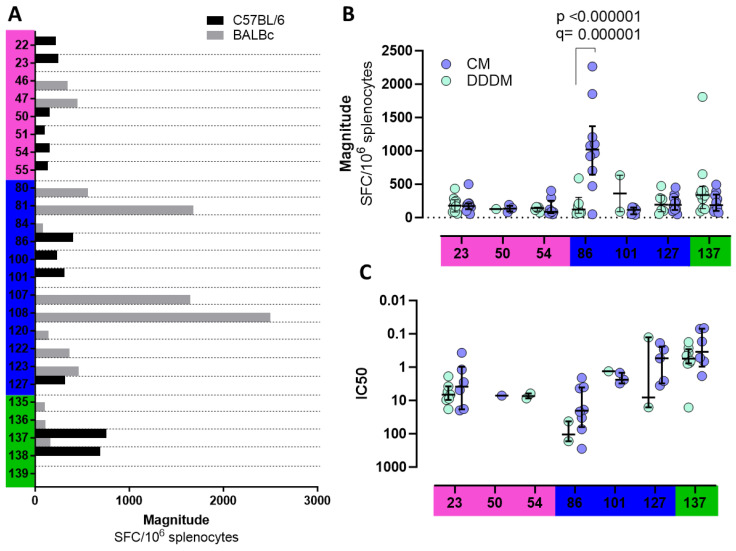
OLP response mapping. (**A**) Responses above the threshold to individual OLPs in C57BL/6 and BALB/c mice vaccinated with the DDDM regimen (**B**) Responses to individual OLPs comparing DDDM with the CM regimen in C57BL/6. Responses between vaccination regimens were compared using multiple *t*-tests with false discovery rate correction. (**C**) IC50 of individual peptides comparing DDDM with the CM regimen in C57Bl/6. IC50s between OLPs were compared using the Kruskal–Wallis test with Dunn’s correction. Statistical significance is shown by *p* and q values.

To explore individual OLP-specific responses, reactive peptide pools after DDDM vaccination in C57BL/6 or BALB/c mice (1D, 1G, 1H, 1K, 1L, 2A, 2B, 2C, 2D, and 2E) were deconvoluted and single reactive OLPs were tested (Figure 5A). Fine mapping of OLP-specific responses identified IFN-γ-mediated responses to a different set of peptides in C57BL/6 compared to BALB/c mice, except for peptide OLP 137 which was targeted in both strains. In C57BL/6 mice vaccinated with DDDM, OLP 137 was the one that triggered the strongest responses, while in BALB/c mice, it was OLP 108.

We then selected OLP-triggering IFN-γ responses in C57BL/6 mice and compared the magnitude of the response to individual peptides between DDDM and CM vaccination (Figure 5B). When responses to two overlapping peptides were observed (e.g., 100 and 101), the one triggering the strongest response was selected. These OLPs were then titrated to determine whether the DDDM and CM vaccination regimens induced responses of different functional avidity (Figure 5C). Notably, the CM regimen triggered responses of significantly higher magnitudes to OLP 86 than the DDDM regimen. In addition, the responses also tended to be of higher functional avidity, although this did not reach statistical significance. Nevertheless, the statistical potency of these experiments was limited by the small number of DDDM-vaccinated cell samples available for titration experiments. When comparing the IC50s between vaccination regimens, OLP 137 was the one with the lowest IC50, indicating a higher affinity of its MHC–OLP–TCR interaction.

### 3.3. T-Cell Responses to HTI Are Maintained over Time

**Figure 6:**
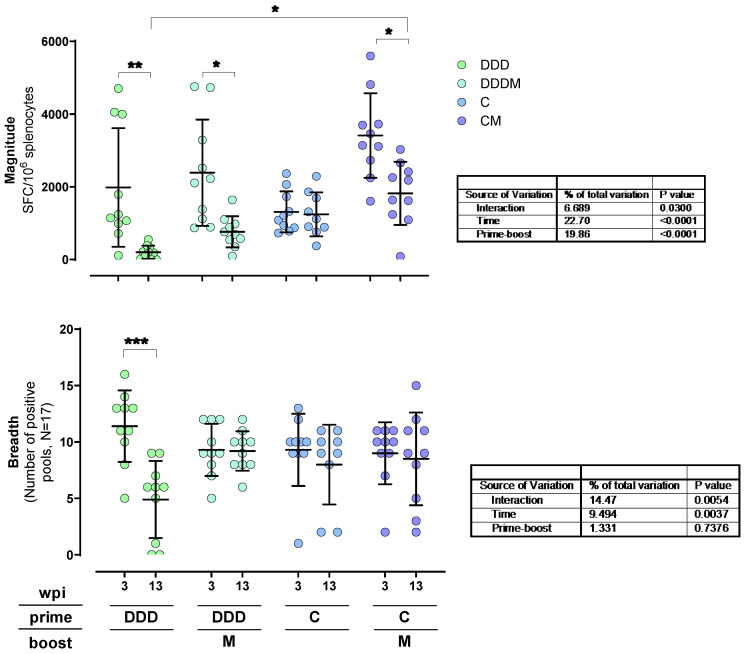
Maintenance of the IFN-γ T-cell response over time. Magnitude (**upper graph**) and breadth (**lower graph**) are shown. Statistically significant differences were tested with a two-way ANOVA and multiple comparisons performed with Tukey’s test. Sources of variation and the *p*-values of the different two-way ANOVA factors are indicated in the table below each graph. Adjusted Tukey’s *p*-values are shown as * *p* < 0.05, by ** <0.01, and by *** <0.001.

We evaluated how responses were maintained 13 weeks post-immunization (wpi) in the four regimens used. Here, IFN-γ T-cell responses to HTI were examined in an additional group of C57BL/6 mice immunized with each of the four vaccination regimens. After 13 weeks, unspecific T-cell responses in the C and CM groups decreased and were comparable to the ones observed in the other regimens (Figure S2). The total magnitude of HTI-specific responses decayed in the DDD, DDDM, and CM groups (adjusted Tukey *p* = 0.0035, 0.0107, and 0.0134, respectively). In the DDD group, a significant reduction in the breadth of the response was also detected (adjusted Tukey *p* = 0.0003). Conversely, for the rest of the groups, the breadth was maintained for 10 additional weeks. Notably, at 13 weeks post-vaccination, the CM regimen was still triggering the highest magnitude responses, which were significantly higher than for the DDD regimen (adjusted Tukey *p* = 0.0111) (Figure 6).

**Figure 7:**
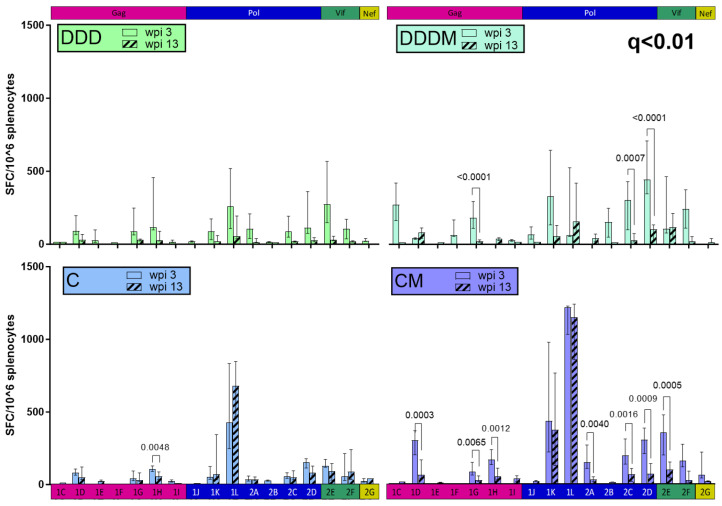
Maintenance of the focus of the response. The responses of the individual pool responses at 3 and 13 wpi were compared using the Mann–Whitney test, with FDR correction for multiple comparisons. Only significant *p*-values (*p* < 0.05) with q < 0.01 are shown.

We also investigated whether the focus of the response changed at 13 wpi (Figure 7). Pool-specific responses in the DDDM and CM groups generally decayed by 13 wpi, reaching statistical significance for several of them (Figure 7). Interestingly, 1K and 1L, the immunodominant pools in the C and CM regimens that contained OLP 86, maintained their high IFN-γ responses at 13 wpi. Therefore, the total magnitude of the response after a C prime was maintained compared to the DDD and DDDM regimens.

## 4. Discussion

Forty years of research have led to the general assumption that an effective HIV vaccine will benefit from the induction of a strong T-cell response, ideally focused on regions of the virus where escape mutations will have high viral replication capacity cost [4]. A combination of different vaccine vectors, expressing the same antigen, in heterologous prime-boost strategies has been shown to increase the response to vaccine immunogens and achieve better protection against infectious diseases [62,65,66]. Accordingly, we tested HTI immunogenicity and immunodominance using three DNA primes or one ChAdOx1 prime, combined with an MVA boost vaccination, in two different mouse strains.

The different vaccination regimens tested caused different levels of unspecific spleen T-cell responses, which were remarkably high when using a ChAdOx1 prime. This was probably caused by a higher capacity of the ChAdOx1 and MVA vectors to directly activate the innate immune system through pathogen-associated molecular patterns (PAMPs) [67]. This higher basal capacity to activate immune cells by the ChAdOx1 vector was also reflected in higher HTI-specific IFN-γ responses in ex vivo analyses. Our data also indicate that ChAdOx1 was a more potent prime for a subsequent MVA boost than three DNA immunizations in terms of magnitude, in two inbred mouse strains with different MHC alleles. However, this increased magnitude was not accompanied by a broader breadth, which was similar between the different immunization regimens and tended to be higher in the three DNA primes. There are very few studies comparing the same vaccination regimens, but in a study comparing DNA plasmid, ChAdOx1, and MVA vaccination against human papillomavirus (HPV), the CM regime triggered the highest responses [68]. Further comparison with other studies is limited by the use of different doses, time between vaccinations, and prime-boost combinations.

Overall, the focus of the response to the different HIV protein fragments that form HTI was not a representation of their percentual contribution to the amino acid sequence. Although 45% of the HTI sequence is derived from Gag, its contribution to the total responses was always lower. On the other hand, responses to Pol and Vif represented a higher percentage of the total magnitude than their contribution to the HTI sequence, at 44% and 8%, respectively. This likely reflects the presence of more murine MHC class I restricted epitopes in these regions than in Gag. Indeed, we found that in C57BL/6 mice, a great part of the increased magnitude of the response to the CM regimen was targeting two Pol-derived peptide pools, 1K and 1L. Remarkably, the 1L pool contained the immunodominant OLP 86. Curiously, this higher magnitude was not correlated with higher affinity as peptide-specific T-cells showed lower functional affinity than other OLP recognized by the same mouse strain. Higher functional affinity of epitope-specific responses has been related to variant cross-reactivity and improved in vivo control of HIV infection [57]. Consequently, these data indicate that selecting vaccination regimens solely on the magnitude of induced responses may not necessarily help to drive responses with the most potent antiviral activity.

We also observed higher overall T-cell responses in BALB/c mice compared to C57BL/6 mice, which was expected since BALB/c mice have six different MHC alleles while C57BL/6 mice only have two. Since these two strains do not share a single MHC allele (C57BL/6: H-2Kb, H-2Db; BALB/c: H-2Kd, H-2Dd, H-2Ld, I-Ad, I-Ed), it was also to be anticipated that they would share few responses to specific pools and single OLPs, showing different immunodominance profiles. Regardless of the different MHC genetics, CM was the best prime-boost regimen in both mice strains, although in C57BL/6 mice, it directed most of the response to a specific, strongly dominant epitope. In BALB/c mice, there were several co-dominant pools targeted across all prime-boost regimens, especially 1K and 2B. In general, the magnitude of the response and the frequency of recognition were related, although some low-magnitude responsive pools (2C, 2D, and 2E in C57BL/6) were frequently targeted. Thus, the immunodominance profiles did not only differ between mouse strains but also depending on the vaccination regimen used. This indicates that immunodominance can be modulated by using different vaccine vectors and vaccination regimens. The mechanisms triggering different immunodominance after vaccination with different vectors are largely unknown and deserve further study. A better understanding of vector-mediated immune modulation will allow a more rational selection of vaccination strategies to better target immune responses.

We also assessed whether there were differences between vaccine regimens and mouse species in terms of the maintenance of HTI-specific responses. In C57BL/6 mice, all responses were reduced at 13 wpi compared to 3 wpi, except for single-ChAdOx1 vaccination where responses were largely maintained at 13 wpi. Immunodominant pools in the CM regime also maintained their magnitude of response at 13 wpi. Interestingly, regimens that included an MVA booster vaccination showed a marked reduction of the overall strongest responses at 3 wpi. Regardless of the reductions in magnitudes, the distribution and focus of the response did not change over time. Further work is needed to understand to what level these differences are related to the induction of memory or self-renewing T-cell responses by one or the other regimen.

## 5. Conclusions

In the last decades, several vaccine vectors have been developed, providing a wide panel of options to develop new vaccines. However, not all vaccine vectors are equally good at triggering T-cell responses and there is a lack of data comparing vaccine vector combinations to guide prime-boost strategy design. With the present report, our aim was to fill the lack of head-to-head comparisons between prime-boost regimens using a DNA plasmid, MVA, and ChAdOx1 vaccine vectors. We showed that the heterologous CM HTI prime-boost regimen triggered the highest magnitude responses in two mouse strains and generated responses that were better maintained over time. In addition, we assessed the capacity of the different vaccination regimens to modulate immunodominance profiles. Our findings suggest that the choice of prime-boost regimens is crucial in determining the magnitude and focus of T-cell responses, providing further guidance in selecting vaccination strategies. Further research will be needed to better understand the mechanisms underlying the differences in T-cell responses caused by the different combinations of vaccine vectors and if they can be generalized to other antigens.

## Data Availability

Data are available from the authors after reasonable request.

## Supplementary Materials

The following supporting information can be downloaded at: https://www.mdpi.com/article/10.3390/vaccines12030279/s1, Table S1: Overlapping peptide pools design, Figure S1: Mean INFγ SFC/10^6^ spleen cells background, Figure S2: Mean INFγ SFC/10^6^ spleen cells background.
