# Supplementary material for: Impact of ChAdOx1 or DNA Prime Vaccination on Magnitude, Breadth, and Focus of MVA-Boosted Immunogen-Specific T Cell Responses
Source: Vaccines (Basel). 2024 Mar 7;12(3):279. doi: 10.3390/vaccines12030279 (PMC10975332; doi:10.3390/vaccines12030279)
Supplement: Supplementary file 1 [file vaccines-12-00279-s001.zip › vaccines-2876420-supplementary.pdf]

# Supplementary Data

## Impact of ChAdOx1 or DNA prime vaccination on magnitude, breadth, and focus of MVA boosted immunogen-specific T cell responses

Alex Olvera[1,2,3,*], Luis Romero-Martin[1,‡], Bruna Oriol-Tordera[1,Ψ], Miriam Rosas-Umbert[1,#], Tuixent Escribà[1], Beatriz Mothe[1,2,3,4,5], Christian Brander[1,2,3,6]

[1] Irsicaixa - AIDS Research Institute, 08916 Badalona, Spain.

[2] Universitat de Vic-Universitat Central de Catalunya (UVic-UCC), Vic, Spain.

[3] CIBERINFEC – ISCIII.

[4] Department of Infectious Diseases, Hospital Germans Trias I Pujol (HUGTIP), Badalona, 08916 Barcelona, Spain

[5] Fundació Lluita contra les infeccions, Hospital Germans Trias I Pujol (HUGTIP), Badalona, 08916 Barcelona, Spain.

[6] Institució Catalana de Recerca i Estudis Avançats (ICREA), Barcelona, Spain.

Actual address:

‡ Institut Pasteur, Paris, France

luis.romero-martin@pasteur.fr

[Ψ] Almirall, Barcelona, Spain

[#] Aarhus University, Aarhus, Denmark

Corresponding author:

[*] Alex Olvera

aolvera@irsicaixa.es

*__Table S1__. Overlapping peptide pools design*

| | | 1C | 1D | 1E | 1F | 1G | 1H | 1I | 1J | 1K | 1L | 2A | 2B | 2C | 2D | 2E | 2F | 2G |
|---|---|---|---|---|---|---|---|---|---|---|---|---|---|---|---|---|---|---|
| **HIV** | **Protein (% in HTI)** | Gag (45%) | | | | | | | Pol (44%) | | | | | | | Vif | | Nef |
| | **Subunit** | p17 | | p24 | | | | p2p7p1p6 | PRT | | RT | | | INT | | Vif | | Nef |
| **Pool** | **ID** | 1C | 1D | 1E | 1F | 1G | 1H | 1I | 1J | 1K | 1L | 2A | 2B | 2C | 2D | 2E | 2F | 2G |
| **HTI** | **Fragment** | 1 | | 2 / 3 | 4 | | 5 / 6 | 7 | 8 | | 9 / 10 | | 11 | 12 | 13 | 14 | 15 | 16 |
| | **Length (AA)** | 78 | | 14 / 11 | 60 | | 14 / 15 | 27 | 55 | | 17 / 55 | | 34 | 34 | 17 | 26 | 19 | 13 |
| | **AA position in HXB2R protein** | 17-50 | 51-94 | 162-170<br>193-203 | 223-245 | 246-282 | 296-308<br>350-363 | 426-452 | 101-121 | 122-155 | 189-205<br>365-372 | 373-419 | 464-496 | 925-958 | 981-996 | 26-50 | 166-183 | 57-68 |
| | **OLP ID – First** | 5 | 16 | 27 | 35 | 43 | 51 | 61 | 69 | 78 | 86 | 96 | 107 | 117 | 127 | 132 | 140 | 146 |
| | **OLP ID – Last** | 15 | 26 | 34 | 42 | 50 | 60 | 68 | 77 | 85 | 95 | 106 | 116 | 126 | 131 | 139 | 145 | 147 |

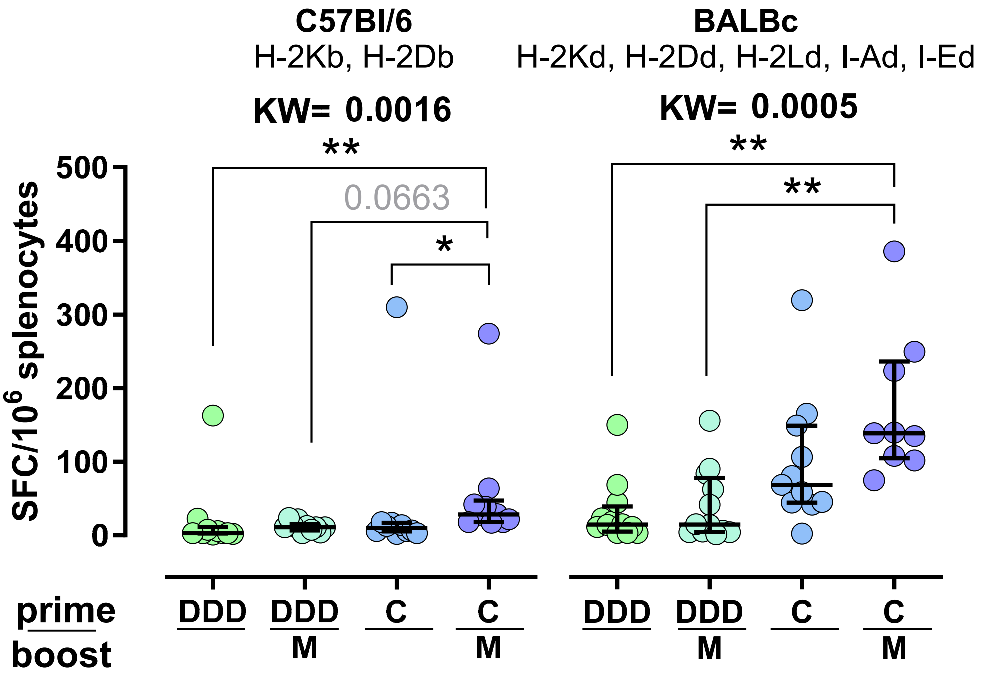


*__Figure S1. Mean INFγ SFC/10[6] spleen cells background.__ Unstimulated negative control triplicates in C57BL/6 and BALBc mice vaccinated with four different prime-boost regimens (DDD, DDDM, C, and CM) are shown. Statistically significant differences (p<0.05) between treatments were tested with the Kruskal-Wallis test with Dunn's correction for multiple comparisons. Significant adjusted Dunn's p values are shown as * p<0.05 by, ** <0.01 by, *** <0.001, and **** <0.0001, trends (p< 0.1) are indicated by grey numbers.*

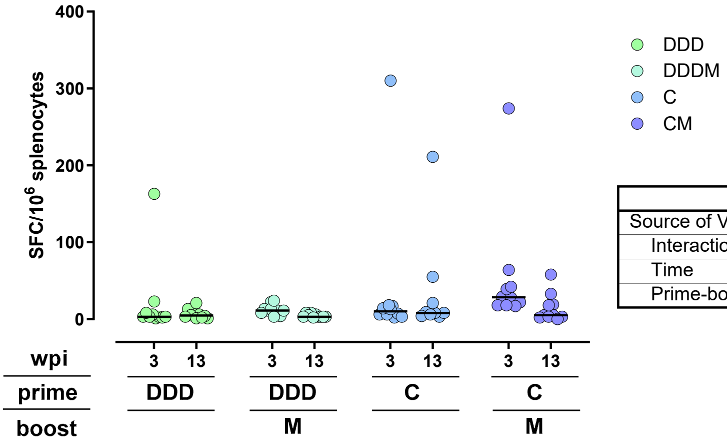


| | | |
|---|---|---|
| Source of Variation | % of total variation | P value |
| Interaction | 1.837 | 0.6944 |
| Time | 2.465 | 0.1670 |
| Prime-boost | 5.839 | 0.2119 |

***Figure S2. Mean INFγ SFC/106 spleen cells background.*** *Unstimulated negative control triplicates in C57BL/6 mice vaccinated with four prime-boost regimens (DDD, DDDM, C, and CM) are shown 3- and 13-weeks post-immunization (wpi). Statistically significant differences (p<0.05) between treatments were tested with a 2-way ANOVA with Tuckey's correction for multiple comparisons. Significant adjusted Tukey's p values are shown as * p<0.05 by, ** <0.01 by, *** <0.001, and **** <0.0001.*
